# Recent Advances in Monoclonal Antibody-Based Approaches in the Management of Bacterial Sepsis

**DOI:** 10.3390/biomedicines11030765

**Published:** 2023-03-02

**Authors:** Kusum Kharga, Lokender Kumar, Sanjay Kumar Singh Patel

**Affiliations:** 1 School of Biotechnology, Faculty of Applied Sciences and Biotechnology, Shoolini University, Solan 173229, Himachal Pradesh, India; 2 Cancer Biology Laboratory, Raj Khosla Centre for Cancer Research, Shoolini University, Solan 173229, Himachal Pradesh, India; 3 Department of Chemical Engineering, Konkuk University, Seoul 05029, Republic of Korea

**Keywords:** monoclonal antibody therapy, sepsis, septic shock, inflammation, bacterial infection

## Abstract

Sepsis is a life-threatening condition characterized by an uncontrolled inflammatory response to an infectious agent and its antigens. Immune cell activation against the antigens causes severe distress that mediates a strong inflammatory response in vital organs. Sepsis is responsible for a high rate of morbidity and mortality in immunosuppressed patients. Monoclonal antibody (mAb)-based therapeutic strategies are now being explored as a viable therapy option for severe sepsis and septic shock. Monoclonal antibodies may provide benefits through two major strategies: (a) monoclonal antibodies targeting the pathogen and its components, and (b) mAbs targeting inflammatory signaling may directly suppress the production of inflammatory mediators. The major focus of mAb therapies has been bacterial endotoxin (lipopolysaccharide), although other surface antigens are also being investigated for mAb therapy. Several promising candidates for mAbs are undergoing clinical trials at present. Despite several failures and the investigation of novel targets, mAb therapy provides a glimmer of hope for the treatment of severe bacterial sepsis and septic shock. In this review, mAb candidates, their efficacy against controlling infection, with special emphasis on potential roadblocks, and prospects are discussed.

## 1. Introduction

The definition of sepsis has been a concern of constant evolution and fine-tuning [1]. With advancing knowledge on the pathogenic mechanism of sepsis, currently “sepsis is well-defined as a serious, potentially life threatening, organic dysfunction initiated by an inadequate or dysregulated host response to infection” [2,3]. According to a global analysis, approximately 48.9 million people are estimated to be affected by sepsis yearly and 11 million deaths are estimated to occur by sepsis [4]. Despite the advances in understanding the pathology of sepsis and the development of its treatments, sepsis remains the leading cause of mortality worldwide [5]. Sepsis is a multifaceted disorder involving inflammation and anti-inflammation disbalance leading to the unregulated widespread release of inflammatory mediators, cytokines, and pathogen-related molecules leading to systemwide organ dysfunction [6]. Although our knowledge of the etiology, pathophysiology, and immunology has improved drastically over the past few years, the knowledge regarding the successful management of the same remains limited [1]. Early diagnosis followed by immediate treatment entails the success rate of treatment in sepsis [7]. Currently, treatment for sepsis and septic shock deeply relies on fluid resuscitation and other general supportive measures along with broad-spectrum antibiotics administration [8].

With the advent of antibiotics in the 20th century, they remain [9] the mainstay for the treatment of bacterial infection [10]. However, empirical use of broad-spectrum antibiotics has been associated with increased mortality and the development of antibiotic resistance [11]. The selective pressure of the unsupervised use of antibiotics has already driven bacteria to develop resistant genes against antibiotics [12]. The rise of antibiotic resistance is notably one of the biggest threats of the 21st century leading to therapeutic failure in the field of infectious diseases [13]. In fact, the dawn of extensively pandrug-resistant (PDR), multidrug-resistant (MDR), and extensively drug-resistant (XDR) strains of ESKAPE pathogens (*Acinetobacter baumannii*, *Enterobacter* spp., *Enterococcus faecium*, *Klebsiella pneumoniae*, *Pseudomonas aeruginosa,* and *Staphylococcus aureus*) combined with the rising gap between the development of antibiotic resistance and novel antibiotics has compelled researchers to shift focus on devising alternative innovations for combating bacterial infection [14]. Of such strategies, monoclonal antibiotics (mAbs) stand out as a promising avenue. Serum treatment was one of the effective ways used to treat bacterial infections early in the 1890s. However, limited spectrum and safety concerns such as allergy and cross-reaction led to its discontinuation [15]. The development of hybridoma technology in 1975 revolutionized the field of research and medicine, garnering the 1984 Nobel prize recognition in physiology and medicine [16,17]. With the introduction of hybridoma technology, mAbs have had a profound impact on immunotherapy, providing large-scale production of pure antibodies with improved specificity and reduced immunogenicity [16]. 

The major obstacle hindering the successful translation of drug candidates in sepsis is the complexity of disease progression that leads to a heterogeneous population with variable underlying clinical presentation, comorbidities, and prognosis abilities [18]. Therefore, monoclonal antibodies targeted against a particular sepsis biomarker present a viable therapeutic option [18]. Monoclonal antibodies may be beneficial in the treatment of sepsis by either directly impeding the growth of the pathogen or by immunomodulation. Initially overshadowed by viral infection and cancer, the application of mAbs in the treatment of bacterial infection is fairly a new approach with only a few FDA-approved drugs [15]. However, it has garnered considerable attention and several promising candidates for mAbs are undergoing clinical trials at present. The present manuscript aims to provide a comprehensive journey of establishing mAbs as a potential alternative therapy for sepsis by highlighting milestone achievements that were ratified by the Federal and Drug Administration (FDA) and those that failed clinical trials but have significantly contributed to our knowledge base.

## 2. Pathophysiology of Sepsis

Sepsis is a multifaceted chaos of wavering balance between inflammation and anti-inflammation leading to the unregulated widespread release of inflammatory mediators, cytokines, and pathogen-related molecules [6]. This dysregulated host response further activates coagulation and complements cascades that often result in death accompanied by multiple organ dysfunction [19].

**Figure 1:**
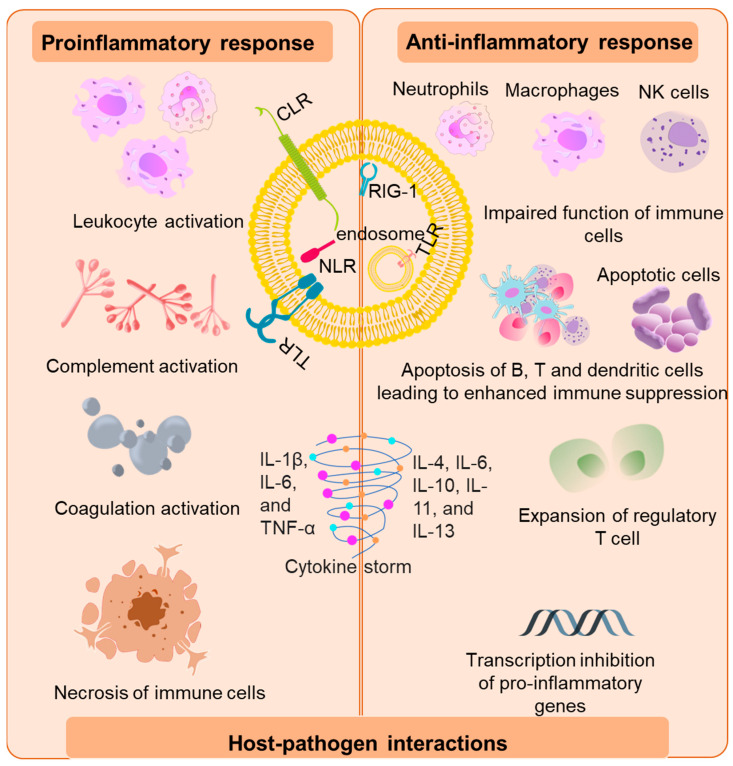
Immunopathogenesis of sepsis. The initial host response is triggered by the binding of bacterial virulence factors to the various pattern recognition receptors such as toll-like receptors (TLR), C-type lectin receptors (CLRs), nucleotide-binding oligomerization domain (Nod)-like receptors and retenoic-inducible gene I (RIG-I). The pro-inflammatory response is marked by the activation of leukocytes which further activates components of the complement and coagulation systems and vascular endothelium via secretion of mediators such as cytokines, reactive oxygen species, and proteases. This led to release of damaged associated molecular patterns (DAMPs) that further exacerbate the pro-inflammatory response. The anti-inflammatory response is represented by impaired function of immune cells, abnormal level of apoptosis, exhaustion of B cells, T cells, and dendritic cells due to negative regulation of TLR signaling and inhibition of pro-inflammatory genes leading to “immunoparalysis”.

The initial activation of the host immune system is mediated by the binding of invading pathogens by pattern-recognition receptors (PRR) on the surface of antigen-presenting cells (APCs) [20]. The PRR-like toll-like receptors (TLR), C-type lectin receptors (CLR’s), retenoic-inducible gene I (RIG-I), and nucleotide-binding oligomerization domain (Nod)-like receptors recognize pathogen-associated molecular patterns (PAMPs) [21,22] and/or host-derived damaged associated molecular patterns (DAMPs) [23]. Murine and mouse models of sepsis revealed that TLR 2, 3, 4, 7 and 9 are involved in the pathogenesis of sepsis by mediating the host’s innate immune response [24,25,26]. Upon recognition of PAMP and damps by TLRs, TLRs initiate transcription of genes involved in inflammation and adaptive immunity through activation of transcription factors such as nuclear factor-κB (NF-κB), activator protein (AP)-1, and mitogen-activated protein kinase (MAPK). This activation of “early genes” leads to the production of pro-inflammatory cytokines such as interleukins IL-8, 12,18, interferons (INFs), and tumor necrosis factor alpha (TNF-α). The pro-inflammatory cytokines thus produced, downstream recruit a torrent of other inflammatory cytokines and chemokines. Components of adaptive immunity are suppressed during the process [27] (Figure 1).

This overwhelming cytokine storm may initially be beneficial in the prognosis of sepsis; however, it ultimately results in progressive organ failure and finally death [6]. In fact, patients in the later stages of sepsis display “immunoparalysis”, a state of chronic immunosuppression [28]. Immunoparalysis is an adjudication of enhanced apoptosis, pyroptosis of immune cells merged with the exhaustion of T cells that marks a patient’s vulnerability to secondary nosocomial infections and reactivation of viruses [18,29,30]. The clinical decline of multiple organs and the development of intravascular thrombosis have been associated with the overproduction or inadequate degradation of immune cell-extracellular traps originally forged to trap and devour pathogens [30,31].

Although sepsis is recognized as an interplay of dysregulated host immune response to infection, clinical representation of the same remains highly individualized making clinical diagnostics a challenge [1]. This may be due to factors such as genetics and the knowledge gap in our understanding of sepsis pathophysiology [32].

## 3. Current Therapeutic Approaches to Treat Sepsis

Current treatment of sepsis consists mainly of antimicrobial and all-purpose supporting treatment [33]. Early diagnosis followed by immediate treatment entails the success rate of treatment in sepsis [7].

### 3.1. Antimicrobial Therapy

Antibiotic treatment still remains the cornerstone of the treatment of bacterial infection despite increasing antibiotic resistance. It has been demonstrated that administering the proper antibiotics during the initial phase (1–3 h window) considerably lowers mortality [34,35,36]. The administration of empirical broad-spectrum antibiotics is the primary strategy employed to contain the bacterial infection up until the active microorganism has been determined [33]. However, knowledge of local epidemiological data and the antibiotic resistance pattern of circulating causative bacteria is of utmost importance for launching an effective antibiotics regime [37,38,39]. Antibiotic treatment choices are limited as a result of the selective pressure caused by the unregulated use of antibiotics, which has caused bacteria to acquire antibiotic-resistance genes [40].

### 3.2. Immunotherapy

With growing antibiotic resistance and the rising gap between the development of antibiotic resistance and novel antibiotics, the focus has been shifted to devising alternative novel innovations for combating bacterial infection. One such strategy is revisiting the antibody-based therapeutic approach which was once deemed harmful.

#### 3.2.1. Immunoglobulins (Ig)

Ig are heterodimeric glycoproteins secreted by differentiated plasma cells and form the part of the natural defense system of the host. They are composed of two heavy (H) and two light chains (L) [41]. Functionally they can be divided into antigen-recognizing variable domains and constant (C) domains involved in activating the complement system and binding Fc regions of other immunoglobulins. Based on the composition of the constant domain, immunoglobulins are defined into five classes: IgM, IgA, IgG, IgE, and IgD. IgG is further subclassified into IgG1, IgG2, IgG3, and IgG4l; similarly, IgA into IgA1 and IgA2. All subclasses each represent unique biological functions [42].

#### 3.2.2. Intravenous Immunoglobulins

Driven by the hypothesis that sepsis-induced immunosuppression could be neutralized by stimulating the immune response or substitution of the individual immune system component, the therapeutic application of polyvalent intravenous immunoglobulins (IVIg) was postulated [43]. The rationale is the ability of the immunoglobulins to (i) recognize and neutralize pathogens and associated toxins, (ii) anti-apoptotic effect on immune cells, and (iii) inhibit transcription [43]. Although the application of IVIg has been found to significantly reduce the sequential organ failure assessment scores (SOFA), apoptosis [44], and disseminated intravascular coagulation (DIC) [45] while increasing the blood level of immunoglobulins [46], it was unable to reduce mortality in sepsis patients [47]. The meta-analysis carried out by Cui and his group in 2019 revealed reduced mortality among adult patients with sepsis by intravenous administration of IgM-enriched immunoglobulin (IVIgGM). However, the treatment effect tended to diminish or become less consistent when the study was narrowed down to a specific indicator of sepsis, invalidating the large-scale use of IVIgGM as a therapeutic treatment [48].

#### 3.2.3. Monoclonal Antibodies

Most therapeutic drugs targeted against sepsis are liable to failure, probably due to the wrong conception that a single therapeutic strategy would suffice to counteract in equal measure all the heterogeneous population with variable clinical presentation, comorbidities, and prognosis abilities [49]. Over the years, studies have been directed toward finding more appropriate biomarkers by conducting more homogeneous sub-population studies in order to develop better targeted drug candidates [50]. As such, monoclonal antibodies (mAbs) targeted against a single target may potentially be the game changer in the treatment of sepsis. With cancer [51] and viral [52,53] infections taking the majority of the limelight, the use of mAbs in the treatment of bacterial infection is still very young with only three FDA-approved drugs so far [15]. Monoclonal antibodies may provide benefits through two major strategies: (1) monoclonal antibodies targeting the pathogen and its components, and (2) mAbs targeting inflammatory signaling may directly suppress the production of inflammatory mediators.

##### Monoclonal Antibodies Targeting Pathogen and Its Components

**Figure 2:**
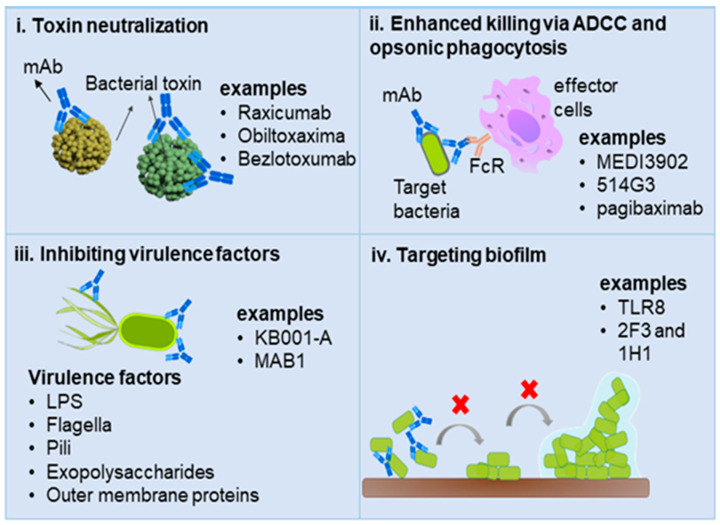
Examples of mAbs that target bacteria with their general mechanism of action.

Monoclonal antibodies can impede bacterial burden via different mechanisms including direct toxin neutralization, inhibiting virulence factors, complement depositions, and enhanced opsonization (Figure 2) [54].

i. Toxin Neutralization

Among the array of virulence factors produced by bacterial pathogens to induce infection, secreted toxins play a pivotal role in the induction of infection in targeted host cells located at the sites of infection or distantly [41]. Numerous pathogenic bacteria such as *Clostridium botulinum*, *Clostridium difficile*, *Clostridium tetani*, *Bacillus anthracis*, *Corynebacterium diphtheriae*, *Vibrio Cholera*, *Escherichia coli*, etc., act by secreting toxins [55,56,57,58,59,60]. Currently, toxins have also been associated with the development of persister cells [61]. Therefore, sequestering toxins though mAbs provides a feasible alternative to reduce infection. In fact, all three FDA-approved mAbs for the treatment of bacterial infections fall under this category.

Raxibacumab (Abthrax^®^), a human mAb raised against the protective antigen (PA) of *Bacillus anthracis*, was approved by the FDA on 14 December 2012 as the treatment therapy for post-exposure inhalation anthrax [62]. During inhalation of anthrax, *B. anthracis* spores deposited in the alveolar spaces of the lungs germinate over a period of 2–43 days into actively dividing bacilli, resulting in bacteremia [63]. Spore germination results in gastrointestinal and oropharyngeal ulceration followed by local edema, necrosis, perforation, and sepsis [64]. These actively dividing bacilli produce tripartite anthrax toxin: (i) protective antigen (PA), (ii) a protease lethal factor (LF), and (iii) an adenylate cyclase edema factor (EF). PA binds to the mammalian cellular receptors and facilitates the translocation of LF and EF into the cells. During the initial stages of infection, the toxins interfere with the signaling pathways and normal functioning of the immune system while the later stage is marked by vascular collapse [65]. Raxibacumab binds to the PA component of the tripartite anthrax toxin and inhibits LF and EF cellular internalization, thereby neutralizing the toxin and preventing the progression of the disease [66]. A study by Cui and his team examined the therapeutic efficacy of raxibacumab in a rat model of anthrax sepsis. Sepsis was first induced using the Bacillus anthracis lethal toxin (LeTx), and raxibacumab was then given post-induction at a dosage of 1–10X of PA. During the beginning of sepsis and 3, 6, 9, or 12 h later, the impact of PA-MAb was examined. Raxibacumab was found to be effective when given to rats for up to 6 h, enhancing their survival and highlighting the efficacy of raxibacumab to manage sepsis [67]. Following further rigorous studies on other animal models [68], raxibacumab was approved for treating inhalation anthrax under the ‘animal rule’ where no other therapeutic option is available [63,66].

Based on the same mechanism of neutralizing anthrax toxin by binding and disarming PA another novel mAb, obiltoxaximab (Anthim^®^), a chimeric mAb was developed by Elusys Therapeutics [69]. Under the US FDA animal rule, in March of 2016, obiltoxaximab in combination with antibiotics was permitted for the treatment of inhalation anthrax where other treatment options are unavailable [69]. The animal-to-human dose was selected and justification was provided by comparing different animal [70] and human studies [71], where obiltoxaximab at 16 mg/kg was found to exhibit a favorable tolerability and safety profile following intramuscular and intravenous exposure. When Yamamoto and his team investigated the impact of a single intravenous or intramuscular dose of 2–16 mg/kg obiltoxaximab at various time intervals relative to *B. anthracis* spore exposure, they showed the effectiveness of obiltoxaximab in toxin neutralization pre- and post-exposure administration [72]. This study showed that obiltoxaximab, when given as a 5-day treatment, was able to protect 89–100% of rabbits either given alone or in combination with levofloxacin 9 h after the challenge. Comparatively, only 33% of rabbits were protected by levofloxacin monotherapy. Coherently, a single intramuscular dose of obiltoxaximab at 16 mg/kg given to cynomolgus macaques prior to the onset of bacteremia resulted in 100% survival when given 1–3 days prior to exposure and 38–100% when given 18–24 h following exposure, demonstrating the efficacy of obiltoxaximab as a potent therapeutic agent by inhibiting bacterial spread, ameliorating toxemia, and promoting the survival of the animals. However, administration of obiltoxaximab after the commencement of bacteremia resulted in lowered survival rate (25–50%).

Bezlotoxumab (Zinplava^®^), a human mAb targeting toxin B of *Clostridium difficile* was approved by the FDA for the treatment of recurrent *C. difficile* infection (CDI) [73]. A single molecule of bezlotoxumab neutralizes toxin B by binding to two homologous epitopes within the N-terminal half of the combined repetitive oligopeptide (CROP) domain present within toxin B through its two Fab regions, inhibiting the binding of toxin B to target colonocytes [74]. During gut infection, the translocation of bacteria and their virulence factors due to diminished vascular permeability has been implicated in the development of sepsis. In a study conducted to evaluate the protective effect of bezlotoxumab against systemic infection in mice models, it was demonstrated that bezlotoxumab administered at a dose of 10 mg/kg before and after 24 h of infection could prevent systemic infection and atrophy of the thymus. Models of mice treated with bezlotoxumab showed normal levels of CD4^+^ and CD8^+^ cells, which otherwise would reflect immune suppression observed during sepsis [75]. During two large randomized, double-blind trials, intravenous infusion of bezlotoxumab at 10 mg/kg dosage exhibited significant benefit in patients with one or more predefined risks such as age, immunocompromise, history of CDI, and severe CDI. However, an inexplicably increased risk of heart failure was observed in subjects with underlying congestive heart failure [76].

ii. Enhanced Killing via ADCC and/or Opsonic Phagocytosis

Most of the time, mAbs are unable to cause direct bacterial killing and depend on the cooperation of the complement system (complement-dependent cytotoxicity—CDC) and/or phagocytic cells (antibody-dependent cellular cytotoxicity—ADCC) [15,77]. A mAb 514G3 eradicates *S. aureus* by enhancing opsonization [78]. *S. aureus* causes life-threatening conditions and is adept at immune evasion [79]. The cell wall protein A (SpA) of *S. aureus* tightly binds to most immunoglobulin subclasses via their Fc region thereby neutralizing the effector function of antibodies [78]. Anti-SpA mAb 514G3 binds specifically to *S. aureus* surface protein SpA and provides a platform for loading phagocytic cells. Phagocytic cells bind to the Fc domain of anti-SpA mAb 514G3 through their Fcγ receptors and clear *S. aureus* [78]. To determine the protective effect of 514G3 in *S. aureus* bacteremia, Varshney and his group established the mouse sepsis model using MRSA. Before MRSA infection, mice models were treated with 10 mg of 514G3, whereas control mice were treated with 10 mg of the VH3/IgG3-k isotype or just the vehicle. On day 14, it was revealed that 60% of mice treated with 514G3 survived, whereas none of the mice in the control group or receiving vehicle alone survived [78].

Similarly, to tackle the clinically important *Pseudomonas aeruginosa,* a bivalent, bispecific human immunoglobulin G1κ monoclonal antibody MEDI3902 biS4aPA was developed. MEDI3902 binds to both the *Pseudomonal* proteins PcrV responsible for host cell cytotoxicity and exopolysaccharide Psl involved in adherence to tissue and colonization [80]. In preclinical studies involving murine and rabbit pneumonia models and in models of thermal and bacteremia, MEDI3902 was demonstrated to reduce bacterial burden, preserve integrity of pulmonary tissue and prevent dissemination of bacteria to the spleen and kidneys [81,82]. MEDI3902 also exhibited a synergistic effect with different antibiotics against different clinical strains of *P. aeruginosa* [81]. In a Phase 1 study conducted in healthy adults, MEDI3902 showed that no treatment-emergent adverse events (TEAEs) were observed [80]. MEDI3902, when administered at 250, 750, and 1500 mg/kg doses, exhibited linear pharmacokinetics whereas dosages between 1500 and 3000 mg exhibited non-linear pharmacokinetics. Serum cytotoxicity antibody and opsonophagocytic killing activity were in correlation to the serum concentration of MEDI3902, encouraging further studies. More recently, the safety, efficacy, and pharmacokinetics of MEDI3902 were evaluated in *P. aeruginosa*-colonized, mechanically ventilated ICU patients [83]. During this randomized double-blind study, pharmacokinetic data showed a low MEDI3902 serum concentration with 500 mg. Patients randomized between 1500 mg and placebo reflected confirmed *P. aeruginosa* pneumonia in 22.4% of patients receiving 1500 mg MEDI3902 and 18.1% of patients receiving placebo. At 21 days post-treatment with 1500 mg, the mean serum concentration of MEDI3902 was 9.46 µg/mL. The study revealed that MEDI3902 was unsuccessful in reducing nosocomial *P. aeruginosa* pneumonia in mechanically ventilated patients.

In a similar account, to minimize the casualty of very-low-birth-weight (VLBW) infants due to *S. aureus*-associated sepsis, pagibaximab, a murine/human chimeric mAb was developed against lipoteichoic acid (LTA) of *S. aureus* [84]. LTA is highly conserved on the cell wall of *S. aureus*, which allows *S. aureus* to evade phagocytosis and sustain bacterial survival by inducing cytokine cascade upon stimulation of TLRs [85]. Pagibaximab was found to be promising in preclinical studies. Pagibaximab, in initial in vitro and in vivo studies, was established to be opsonic, promote phagocytosis, and inhibit the activation of cytokine cascade. However, the Phase III clinical trial, most conclusively, demonstrated a non-significant reduction in sepsis by pagibaximab compared to placebo [86]. The failure of pagibaximab in preventing staphylococcal infection was attributed to the unavailability of LTA for the binding of the mAb (LTA may be present beneath the surface of the cell wall in some *S. aureus* isolates) [87]. The failure of pagibaximab highlighted the importance of selecting the target and the appropriate animal model [86].

iii. Inhibiting Virulence Factors

Bacterial growth and survival can be directly hindered by mAbs targeted against the virulence factors present on the surface of the bacteria. KB001-A is PEGylated mAb fragment directed against the Type III secretion system (TTSS) of *P. aeruginosa* [88]. *P. aeruginosa* is an avid opportunistic pathogen that is generally found to inhabit the airways of lungs affected by cystic fibrosis (CF) [89]. TTSS of *P. aeruginosa* facilitates the release of exotoxins into the cytoplasm of host cells and extracellular spaces, leading to the cytotoxicity of *P. aeruginosa* in host epithelial cells, neutrophils, and macrophages [90]. KB001-A is a modified, recombinant, anti- *P. aeruginosa* PcrV Fab’ antibody that inhibits the function of TTSS by selectively binding to the PcrV [91]. PcrV is a protein located at the tip of TTSS that is crucial for the transport of *Pseudomonal* exotoxins into the host cells [92]. A study conducted to evaluate the safety efficacy, pharmacodynamics, and pharmacokinetic (PK) properties of KB001 in 27 CF patients with chronic *P. aeruginosa* infection revealed that KB001-A had a satisfactory safety profile with an average serum half-life of 11.9 days [91]. No significant difference was observed between the KB001-A treated patients when compared to placebo subjects. However, the 28th day revealed a reduction in the sputum myeloperoxidase, IL-8, IL-1, elastase, and neutrophil counts in the group treated with KB001-A at 10 mg/kg in a dose-dependent manner, indicating a viable alternate option to antibiotic treatment.

Outer membrane proteins (OMPs) are a major source of virulence in gram-negative bacteria making them a potential candidate for the development of mAb [93,94,95,96,97]. Located abundantly on the outer membrane, OMPs are proteinaceous components mostly composed of β-barrel [98]. Proper folding and integration of these β-barrel proteins are highly essential for the viability and pathogenesis of gram-negative bacteria. It is catalyzed by the β-barrel assembly machinery (BAM) [99]. Recent studies reveal that mAb, MAB1, raised against BamA, the central component of the BAM complex was able to retard *E. coli* growth by exerting direct bactericidal activity [100,101]. MAB1 binds to and interferes with the protein folding activity of BamA.

iv. Targeting the Biofilm

Biofilm is the most powerful armor forged by bacteria to survive hostile environments [102]. During biofilm formation, the planktonic bacteria enter a sessile mode of lifestyle [103]. Matrix-encased biofilm insulates bacteria and aids in escaping the deleterious effect of immune macrophages and antibiotics and is thus the reason for chronic infection [104]. Some bacterial populations within the biofilm develop to tolerate higher levels of antibiotics and are called persister cells [105]. Following the erosion of biofilm, these persister cells resume planktonic lifestyles, translocate to different areas, colonize them, and restore the original bacterial population [106]. Therefore, biofilms providing an unlimited reservoir of bacteria have emerged as an attractive target for mAbs.

Extracellular DNA (eDNA) is one of the key components of biofilm and serves as a scaffold for the other biofilm components [107]. eDNA in turn is held in place by various bacterial secreted proteins, most prominent belonging to the DNABII family consisting of an integration host factor (IHF) and histone-like (HU) proteins that are conserved among both gram-negative and gram-positive bacteria. Both of these proteins bind to eDNA in a nonspecific manner. Antibody labeling revealed that the IHF was located at the anchoring nodes in the matrix [108]. Therefore, a native human monoclonal antibody TRL1068 was developed that could disrupt the biofilm by binding to and sequestering the scaffold proteins DNABII [109]. In vitro studies revealed TRL1068 could disrupt the established biofilm at 1.2 µg/mL over 12 h. In vivo efficacy of TRL1068 was established in animal models infected with antibiotic-resistant of *S. aureus* and *Acinetobacter baumannii*, where TRL1068 was demonstrated to significantly reduce mature biofilm in combination with antibiotics daptomycin, vancomycin, and imipenem [109,110], encasing the potential use of TRL1068 as adjunct therapy against difficult-to-treat bacterial infections. Currently, a clinical trial is recruiting patients for the evaluation of safety and efficacy in prosthetic joint infections.

The efficacy of mAb in disrupting biofilm was further investigated by Jurcisek and his group [111]. In this study, two mAbs, one raised against DNABII protein and another against type IV pilus (T4P) of non-typeable *Haemophilus influenzae* (NTHI), were tested to disrupt biofilms composed of two different genera of bacteria, wherein NTHI was allied with another clinically important respiratory tract pathogen (*Burkholderia cenocepacia*, *S. aureus*, *P. aeruginosa*, *S. pneumoniae*, or *Moraxella catarrhalis*). These monoclonals were tested individually as well as in the form of a cocktail. The study revealed that mAb against NTHI type IV pilus (T4P) was effective only against biofilms formed by single species of NTHI and not on biofilms formed by other species singly. Nonetheless, NTHI-directed mAbs were able to disrupt biofilms composed of two different genera of species. On the contrary, mAbs against DNABII protein were adept at disrupting both multi-species biofilms and all single-species biofilms. The highest release of pathogens following the disruption of multi-species biofilms was achieved by a 1:1 cocktail of both mAbs. This study marked the potential use of a mAb cocktail as an alternate therapy to bacterial infections.

Another study highlighting the efficacy of mAb in inhibiting the biofilm of *Staphylococcus epidermidis* was demonstrated by Lyu et al. [112]. YycFG, a two-component signal transduction system (TCS), is pivotal for biofilm formation in *S. epidermidis* [113]. During the study, Lyu et al. evaluated the efficacy of mAbs 2F3 and 1H1, in preventing *S. epidermidis* biofilm formation. mAbs 2F3 and 1H1 are directed against the histidine kinase YycG extracellular domain (YycG_ex_) [112]. It was revealed that these mAbs were able to inhibit *S. epidermidis* biofilm formation in a dose-dependent manner. 2F3 and 1H1 administered at a concentration of 120 µg/mL were able to reduce 78.3% and 93.1% biofilm, respectively, as compared to the normal mouse IgG control. Treatment with mAbs 2F3 and 1H1 also exhibited reduced initial adherence and synthesis of polysaccharide intracellular adhesin. Further, a marked reduction in the transcription level of genes responsible for encoding proteins involved in *S. epidermidis* biofilm formation was observed. This study is an indicator of the YycGex domain as a potential candidate for vaccine development to prevent *S. epidermidis* biofilm infections.

##### mAbs Targeting Inflammatory Signaling to Suppress the Production of Inflammatory Mediators and Control Sepsis Progression

**Figure 3:**
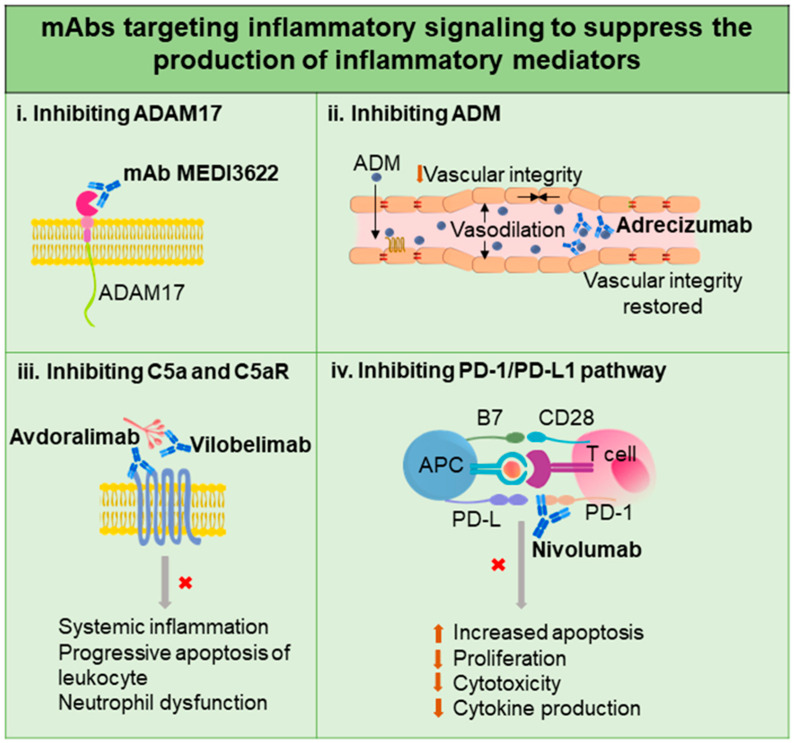
Immunomodulatory targets for the development of mAb to control the progression of sepsis.

As mentioned in earlier sections, sepsis is the result of unregulated hyperinflammation and immune suppression in response to bacterial infections. Therefore, controlling the intermediate mediators of the inflammatory pathway poses an appealing target for developing mAb as a therapeutic agent against sepsis (Figure 3).

During sepsis, neutrophils play a crucial role as first responders; however, they are rendered highly dysfunctional [114]. ADAM17, a transmembrane protease belonging to a disintegrin and metalloproteinase family, is involved in the regulation of various signaling pathways [115]. By conditionally knocking out ADAM17, it was established that the excessive ADAM17 activity in leukocytes has a negative effect on the host response [116]. With the predetermined efficacy of ADAM17 knocking out leukocytes in increasing neutrophil recruitment and reducing bacterial spread during polymicrobial sepsis [117], Mishra et al. investigated the efficacy of ADAM17 mAb MEDI3622 against a murine model of polymicrobial sepsis [118]. This work demonstrated significant sepsis survival following delivery of ADAM17 mAb MEDI3622 both prior to and following infection induction, establishing ADAM17 as the prospective therapeutic target for sepsis control. ADAM17 mAb MEDI3622 also improved survival when administered in combination with antibiotics.

Immunoparalysis due to heightened induced cell death is the hallmark of later stages of sepsis and therefore immunostimulation may prove to be beneficial in controlling sepsis [20]. Following the success in the treatment of cancer, recently there has been an increasing interest in immune checkpoint blockers (ICBs) as candidates for the treatment of bacterial sepsis [119]. In cancer, following the encounter with a foreign antigen, the cytotoxic T lymphocytes (CTLs) expand vigorously [120]. After resolving the inflammation and antigen clearance, the programmed cell death protein 1 (PD-1) receptors on the surface of cytotoxic lymphocytes bind to their ligands, PD-1L and PD-L2, generating negative co-stimulatory signals in order to suppress the CTL expansion [120]. Several studies have documented the enhanced expansion of PD-1 during sepsis. Subjects who died from multiple organ failure resulting from sepsis exhibited increased PD-L1 on macrophages and other APCs [121]. Additionally, a gradual increase in the level of PD-L1 was also seen in monocytes based on the increasing severity of sepsis, supporting the idea that unusual activation of the PD-1/PD-L1 pathway is a major cause of immunoparalysis in sepsis patients [122,123]. Nivolumab is a mAb targeting drug that has proven to be successful at immunomodulation during the treatment of cancer [124]. Nivolumab acts by blocking PD-1 inhibitory pathways, allowing T cells to proliferate. Watanabe et al. found that a single dose of 960 mg of nivolumab was well tolerated and sufficient for maintaining blood concentration levels of nivolumab in a multicenter, open-label phase ½ research on patients with sepsis-induced immunosuppression [125]. Nivolumab 480 mg and nivolumab 960 mg both enhanced immune system indices over time. Additionally, nivolumab and meropenem combination therapy demonstrated that early administration of nivolumab at 6 mg/kg can improve bacterial sepsis when lone antibiotics fail [119], emphasizing the significance of using accurate biomarkers to categorize patients and developing precision medicine in sepsis.

Adrenomedullin (ADM), a 52 amino acid-containing peptide, is required for regulating the endothelial barrier function and vascular tone [126]. Vascular leakage and vasodilation are crucial in the progression of septic shock, where leakage of the vascular membrane is attributed to disrupted endothelial integrity [127]. The level of ADM has been found to rise during sepsis and correlate with the severity of sepsis. Adrecizumab (HAM 8101) is a non-neutralizing mAb directed against the ADM N-terminal that inhibits ADM activity partially. Adrecizumab acts by suppressing the level of ADM in blood concentration. Preclinical studies in the sepsis model demonstrated the efficacy of adrecizumab in improving the endothelial barrier function and reducing mortality induced by sepsis-associated organ failure [128,129]. Following the success in the preclinical trial, Laterre et al. evaluated the safety of adrecizumab by conducting a double-blind, randomized biomarker-guided human trial. During the study, subjects were randomly allocated in a 1:1:2 ratio depending on adrecizumab dosage (adrecizumab 2 mg/kg, 4 mg/kg, and placebo) [130,131]. It was revealed that both doses were well tolerated with no overt signs of harm.

Anaphylatoxins C3a, C4a, and C5a are upregulated due to complement activation during the initial stages of sepsis. Of these, C5a is of particular importance as it possesses pro-inflammatory and chemotactic properties, is spasmogenic, and its sustained level in serum leads to excessive inflammation in sepsis. Disproportionate activation of C5a in sepsis exacerbates systemic inflammation, and leads to apoptosis of immune cells and neutrophil exhaustion, making C5a an attractive pharmacological target. The mouse model of sepsis unveiled the therapeutic potential of blocking C5a or C5aR, where it was revealed that the C5ar1-deficient mice were able to significantly survive mild to moderate sepsis. The decreased mortality was directly associated with improved pathogen clearance and preserved liver function. C5ar1-deficient mice were also shown to have an increased level of pro-inflammatory interferon γ and decreased level of anti-inflammatory cytokine IL-10, validating the role of C5a as a mediator of immunosuppression observed during sepsis.

Vilobelimab (IFX-1) is a chimeric mAb of the IgG4 class developed against the human complement component C5a. A multicenter, randomized, placebo-controlled, double-blind, phase-2a trial involving adult patients suffering from severe sepsis or septic shock exhibiting early onset of infection-associated organ dysfunction was conducted by Bauer et al. in eleven multidisciplinary ICUs across Germany. The study disclosed that subjects displaying severe sepsis and septic shock upon receiving vilobelimab treatment selectively neutralized C5a in a dose-dependent manner exclusive of interference with the normal formation of the membrane attack complex (MAC) nor safety issue. Another mAb avdoralimab directed against C5aR1 inhibits the signaling of C5a via its receptor. A recent study by Carvelli et al. in a randomized controlled trial in patients with COVID-associated pneumonia demonstrated no significant improvement in patients following treatment with avdoralimab [132].

##### Antibody-Antibiotic Conjugate

**Figure 4:**
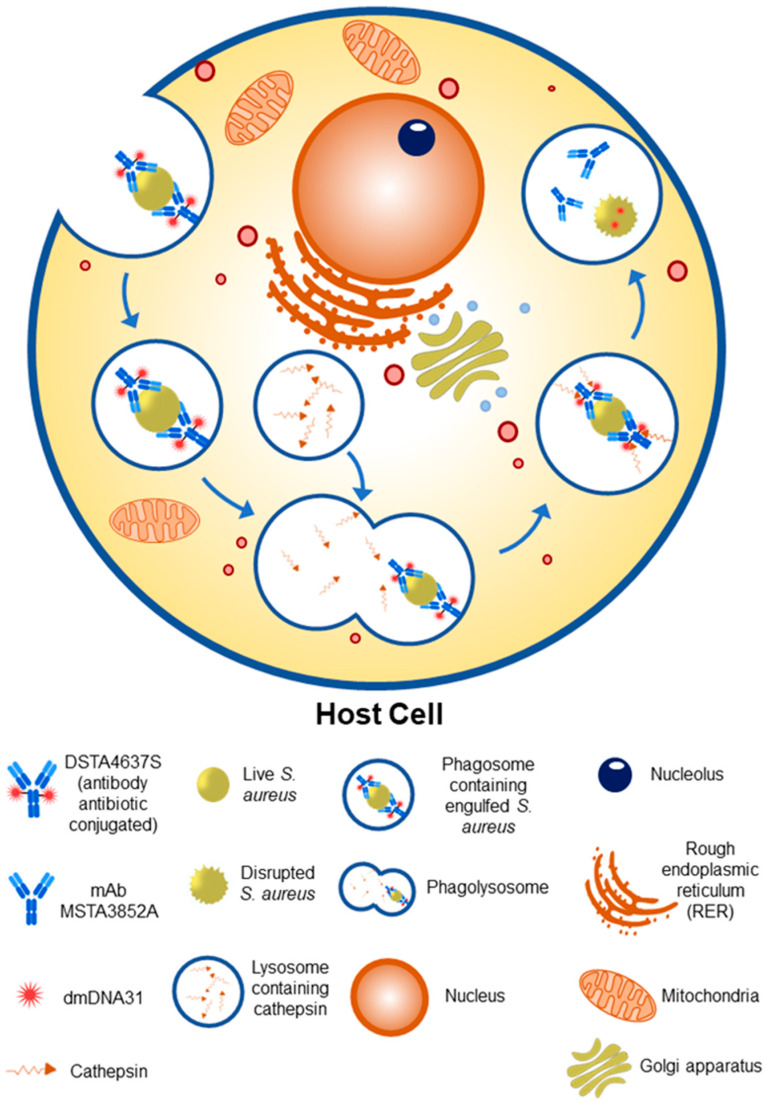
Mechanism of killing intracellular *S. aureus* by DSTA4637S. DSTA4637S binds to *S. aureus*, enhancing phagocytosis. Following internalization by host cells, it fuses with the phagolysosome where lysosomal cathepsins cleave the vc linker releasing the antibiotic dmDNA31. dmDNA31 is responsible for killing the intracellular pathogen.

Another prospective application of mAbs in the treatment of bacterial infection is through the targeted delivery of antibiotics. The AAC is drafted based on the design of antibody-drug conjugates (ADC) that has previously contributed significantly to the treatment of cancer [133]. AAC consists of a mAb directed against a selective target attached to an antibiotic payload through a cleavable and non-cleavable linker [134]. This innovative approach takes advantage of the selectivity and safety of the antibody to administer more potent antibiotics with reduced off-targets. A successful formulation developed based on this mechanistic is DSTA4637S or THIOMAB™, directed against *S. aureus*. DSTA4637S is an AAC containing an engineered human IgG1 anti-*S. aureus* mAb MSTA3852A explicitly for O-Antigen bacterial LPS, and a novel rifamycin antibiotic, dmDNA31 (4-dimethylamino piperidino- hydroxybenzoxazino rifamycin), conjugated through a valine-citrulline (vc) linker cleavable by a protease. DSTA4637S-bound *S. aureus* upon ingestion by phagocytic cells is cleaved through intracellular cathepsin releasing dmDNA31 that kills intracellular *S. aureus* [135] (Figure 4), providing hope for the treatment of intracellular pathogens. Overall, mAbs can potentially treat bacterial infections with a few FDA-approved drugs (Table 1).

## 4. Conclusions

Despite advancements made in the field of science and technology, bacterial sepsis and septic shock remain major health concerns claiming millions of lives every year all over the world. Current therapeutic strategies for treating sepsis still consist of early administration of antibacterial drugs and a general support system aimed towards the restoration of the patient’s hemostasis. Defense against bacterial infection has been dominated by the use of small molecules such as antibiotics over the past decades. However, the escalation of antimicrobial resistance has dwindled the therapeutic efficacy of the antibiotic arsenal to a great extent mandating venture for alternate strategies. Fueled by success stories in the treatment of cancer, mAb has garnered significant attraction as a viable alternative therapy to antibiotics. mAb offers several advantages over antibiotics such as target specificity, reduced immunogenicity, longer half-life, and low probability of developing resistance, making it a plausible alternate therapy for the treatment of sepsis. However, the implementation of mAbs in combating bacterial sepsis has been a rough journey with many pitfalls. With the majority of candidates being inept in clinical translation despite successful preclinical animal studies, mAbs as the treatment of sepsis is still far from being realized. The clinical translation of mAbs in sepsis is also limited by the lack of knowledge regarding the underlying complex physiopathology of the disease itself. Regardless of the increasing understanding of the etiology and pathophysiology of sepsis, researchers are still unable to narrow down the targets for the efficient development of mAbs among the heterogeneous population. Moreover, because mAbs are target specific and need accurate pathogen identification to exert their therapeutic effect, using mAbs in the first hour after exposure is questionable. Furthermore, the limitation of successful clinical translation can be attributed to the lack of an animal model that truly represents the sepsis progression in humans. The safety concerns involving mAbs also remain debatable. The development of mAbs is an expensive process requiring huge investments. Despite the aforementioned drawbacks, mAbs hold huge therapeutic potential as was evidenced by their success in battling cancer. With respect to treating bacterial infection, the approval of three mAbs formulations; raxibacumab, obiltoxaximab, and bezlotoxumab, represents a tiny step toward realizing the therapeutic potential of mAbs against sepsis, encouraging further investigation into this arena.

## Figures and Tables

**Table 1:** Monoclonal-antibody-based biologics that are FDA approved or in clinical trials for the treatment of sepsis.

Name	Target	Target Bacteria	Class of Immunoglobulin	Mechanism of Action	Phase	Trial ID	References
Monoclonal antibodies targeted against the pathogen and pathogen-associated factors							
**514G3**	*Staphylococcus aureus* Protein A (SpA)	Human	IgG3	Enhanced opsonization	Phase II	NCT02357966	[78]
**Raxibacumab**	*Bacillus anthracis* toxin	Human	IgG1	Toxin neutralization	Approved	NCT02016963	[63,66,68]
**Bezlotoxumab** **(Zinplava^®^) (MK-6072, MBL-CDB1, or MDX-1388).**	*C. difficile* toxin B	Human	IgG1	Toxin neutralization	Approved	NCT05304715	[73,74,136]
**MEDI4893 Suvratoxumab**	*Staphylococcus aureus* Alpha toxin (AT)	Human	IgG1	Toxin neutralization	Phase III	NCT05331885	[137,138,139]
**TRL1068**	DNABII proteins such as integration host factor (IHF) and histone-like (HU) proteins of both gram-negative and gram-positive bacteria	Human	IgG	Biofilm disruption	Preclinical/Phase I	NCT04763759	[107,109,110]
**Panobacumab**	LPS O-polysaccharide moiety of *P. aeruginosa* O11	Human	IgM/κ	Complement-dependent enhanced opsonophagocytosis	Phase II	NCT00851435	[140,141]
**ASN100** **Combination of ASN-1 and ASN-2**	*Staphylococcus aureus* alpha-hemolysin (Hla) and leukocidins LukSF-PV, HlgAB, HlgCB, LukED, and LukGH (LukAB)	Human	IgG1/κ	Toxin neutralization	Phase II, terminated	NCT02940626	[142,143]
**Obiltoxaximab**	*Bacillus anthracis* (Protective antigen—PA)		IgG1	Toxin neutralization	Approved	NCT03088111	[69,71,144,145]
**Tosatoxumab** **(Salvecin)** **AR-301**	*Staphylococcus aureus* alpha toxin	Human	IgG1 ʎ	Toxin neutralization	Phase III	NCT03816956	[146]
**Pagibaximab-BSYX-A110**	*Staphylococcus epidermidis* (Lipoteichoic acid—LTA	Mouse/Human chimeric	IgG	Enhanced opsonization	Phase II	NCT00631800	[85,86]
**KB001-A**	*Pseudomonas aeruginosa* Type III secretion system	Recombinant	IgG lacking Fc region	Inhibiting the activity of TTSS	Phase II	NCT01695343	[88,91]
**MEDI3902, Gremubamab**	*Pseudomonas aeruginosa* PcrV protein and Psl exopolysaccharide	Human bispecific	IgG	Complement-dependent enhanced opsonophagocytosis	Phase I	NCT02255760	[83]
Monoclonal antibodies targeting inflammatory signaling that directly suppress the production of inflammatory mediators							
**MEDI3622**	ADAM17 transmembrane protease	Human	IgG1	ADAM17 function-blocking	Preclinical		[115,118]
**Nivolumab** **(opdivo** **^®^** **)**	Protein Domain 1 (PD-1)	Human	IgG4	Inhibiting PD-1/PD-L1 pathway	Phase I	NCT02960854	[119,125,147]
**Adrecizumab**	Adrenomedullin (ADM)	Humanized	IgG1/κ	Blocking adrenomedullin (ADM)	Phase I	NCT03083171	[131,148,149]
**Vilobelimab (IFX-1** **)**	C5a	Chimeric	IgG4	Blocking C5a	Phase II	NCT02246595	[150,151]

## Data Availability

Not applicable.
